# Characterization of a Novel Orbivirus from Cattle Reveals Active Circulation of a Previously Unknown and Pathogenic Orbivirus in Ruminants in Kenya

**DOI:** 10.1128/msphere.00488-22

**Published:** 2023-02-16

**Authors:** Dorcus C. A. Omoga, David P. Tchouassi, Marietjie Venter, Edwin O. Ogola, Solomon Langat, Caroline Getugi, Georg Eibner, Anne Kopp, Inga Slothouwer, Baldwyn Torto, Sandra Junglen, Rosemary Sang

**Affiliations:** a International Centre of Insect Physiology and Ecology, Nairobi, Kenya; b Zoonotic Arbo and Respiratory Virus Research Program, Centre for Viral Zoonoses, Department of Medical Virology, Faculty of Health, University of Pretoria, Gezina, South Africa; c Institute of Virology, Charité Universitätsmedizin Berlin, Corporate Member of Free University Berlin, Humboldt-University Berlin, and Berlin Institute of Health, Berlin, Germany; d Centre for Virus Research, Kenya Medical Research Institute, Nairobi, Kenya; University of Michigan

**Keywords:** orbivirus, arbovirus, cattle, Kenya, Africa

## Abstract

Arboviruses are among emerging pathogens of public and veterinary health significance. However, in most of sub-Saharan Africa, their role in the aetiologies of diseases in farm animals is poorly described due to paucity of active surveillance and appropriate diagnosis. Here, we report the discovery of a previously unknown orbivirus in cattle collected in the Kenyan Rift Valley in 2020 and 2021. We isolated the virus in cell culture from the serum of a clinically sick cow aged 2 to 3 years, presenting signs of lethargy. High-throughput sequencing revealed an orbivirus genome architecture with 10 double-stranded RNA segments and a total size of 18,731 bp. The VP1 (Pol) and VP3 (T2) nucleotide sequences of the detected virus, tentatively named Kaptombes virus (KPTV), shared maximum similarities of 77.5% and 80.7% to the mosquito-borne Sathuvachari virus (SVIV) found in some Asian countries, respectively. Screening of 2,039 sera from cattle, goats, and sheep by specific RT-PCR identified KPTV in three additional samples originating from different herds collected in 2020 and 2021. Neutralizing antibodies against KPTV were found in 6% of sera from ruminants (12/200) collected in the region. *In vivo* experiments with new-born and adult mice induced body tremors, hind limb paralysis, weakness, lethargy, and mortality. Taken together, the data suggest the detection of a potentially disease-causing orbivirus in cattle in Kenya. Its impact on livestock, as well as its potential economic damage, needs to be addressed in future studies using targeted surveillance and diagnostics.

**IMPORTANCE** The genus *Orbivirus* contains several viruses that cause large outbreaks in wild and domestic animals. However, there is little knowledge on the contribution of orbiviruses to diseases in livestock in Africa. Here, we report the identification of a novel presumably disease-causing orbivirus in cattle, Kenya. The virus, designated Kaptombes virus (KPTV), was initially isolated from a clinically sick cow aged 2 to 3 years, presenting signs of lethargy. The virus was subsequently detected in three additional cows sampled in neighboring locations in the subsequent year. Neutralizing antibodies against KPTV were found in 10% of cattle sera. Infection of new-born and adult mice with KPTV caused severe symptoms and lead to death. Together, these findings indicate the presence of a previously unknown orbivirus in ruminants in Kenya. These data are of relevance as cattle represents an important livestock species in farming industry and often is the main source of livelihoods in rural areas of Africa.

## INTRODUCTION

Orbiviruses are arthropod-borne viruses (arboviruses) that are commonly transmitted by mosquitoes, sandflies, biting midges, and ticks (1–4) to a wide range of vertebrates (2, 5–11). The genus *Orbivirus* (family *Sedoreoviridae*, order *Reovirales*) consists of 22 classified virus species (1, 2). About 15 potential orbivirus species await classification by the International Committee on Taxonomy of Viruses (ICTV). The virion of orbiviruses is nonenveloped, icosahedral, and has a triple-capsid structure. The genome is composed of 10 linear segments of double-stranded RNA (dsRNA). The segments encode seven structural proteins (virion proteins VP1 to VP7) and five nonstructural proteins (NS1 to NS5) (1, 5, 12). Encoded proteins of these segments vary between viruses of different species and the pattern of segment-encoded proteins is linked to the arthropod vector (13). The VP2 (OC1) and the VP5 (OC2) proteins form the outer capsid and mediate serological reactivity. The VP3 (T2) protein forms the inner capsid which controls the size and organization of the capsid structure and interacts with internal proteins (1). VP3 (T2) is either encoded by segment 2 in tick- and mosquito-borne orbiviruses or segment 3 in culicoides-borne orbiviruses (14–18). In some studies, the genome segment has been used for classification rather than the encoded protein, resulting in some level of nonuniformity and confusion (14). The intermediate capsid protein VP7 (T13) is widely used for virus serotype and species-specific identification (15, 19). Orbivirus genome segments have terminal noncoding regions (NCRs) which are partially conserved within species (15, 18).

The genus contains several viruses that can cause severe disease in wild and domestic animals, such as Bluetongue virus (BTV), African horse sickness virus (AHSV), and Epizootic hemorrhagic disease virus (EHDV). BTV mainly causes disease with high mortality in sheep but can also infect cattle, goats, buffalo, antelope, deer, elk, and camels (20–22). AHSV can lead to fatal disease in horses and mules and also infects donkeys, zebras, and other equines. EHDV mainly infects wild and domesticated ruminants causing hemorrhagic disease in deer while domestic ruminants are subclinically infected.

Orbiviruses are distributed worldwide and their emergence and distribution depend on the abundance of competent vectors and environmental conditions that favor virus transmission between vectors and hosts (23–25). In Kenya and East Africa, the most common orbiviruses are the culicoides-borne BTV that causes Bluetongue disease (BT) and AHSV that causes African horse sickness (AHS), both notifiable diseases according to the World Organization of Animal Health (WOAH). BTV has been reported in different parts of Kenya in various livestock species, including sheep, goats, cattle, and camel with high prevalence rates of up to >80% signifying widespread exposure (26–28). AHSV has been found in donkeys and horses in Kenya (29).

In this study, we report the discovery and molecular and phenotypic characterization of a novel orbivirus designated Kaptombes virus (KPTV) from a diseased cow with evidence of circulation in indigenous cattle and local breeds of small ruminants in Kenya.

## RESULTS

### Kaptombes virus induces cytopathic effect in Vero CCL-81 cells.

**FIG 1:**
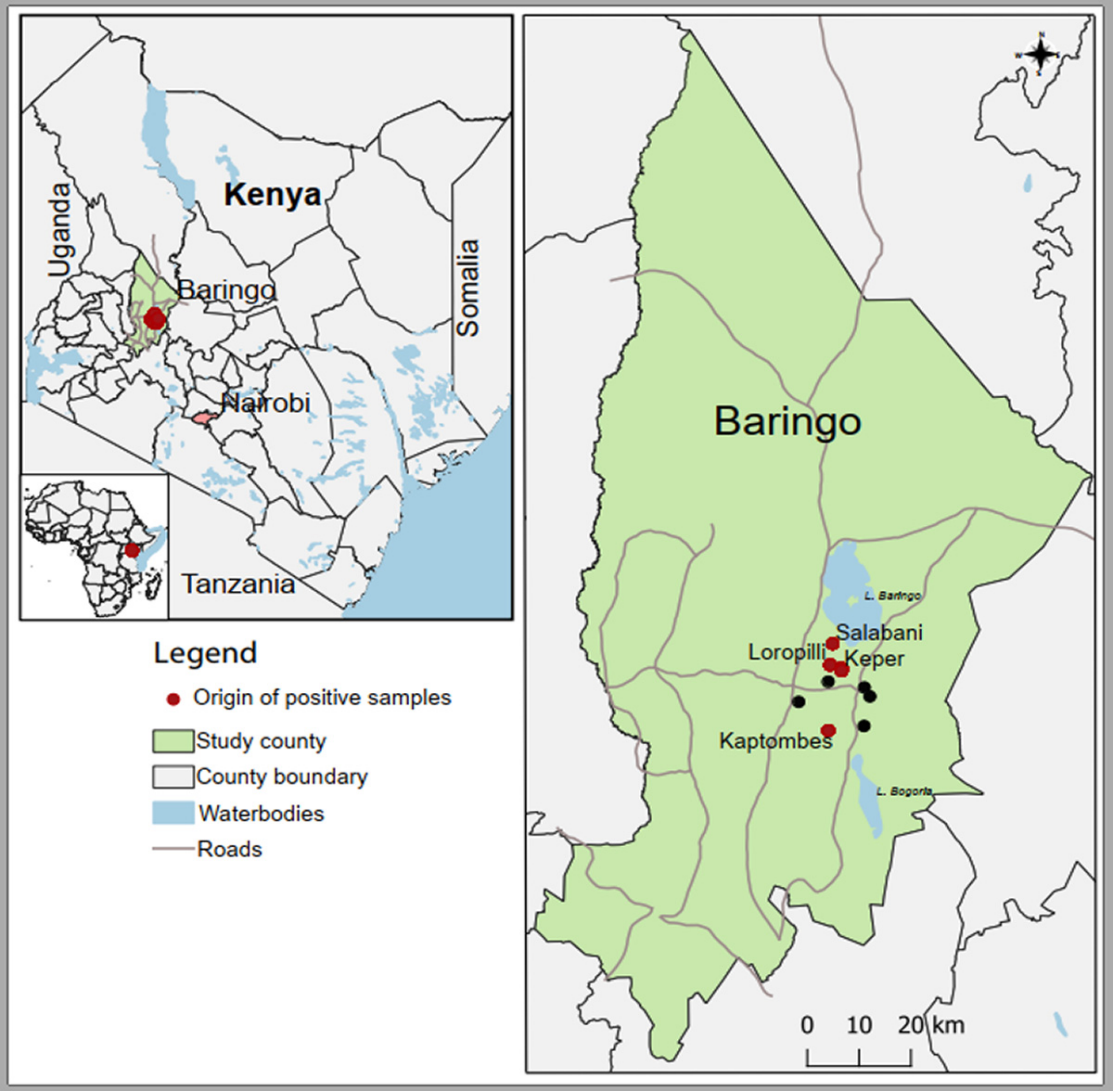
A map of Kenya showing the sampling sites within Baringo county. Red spots represent sites from where positive samples originate. Black dots represent other sampling sites. The maps were created in the open source GIS software, QGIS 3.22 using GPS coordinates and shape files derived from Natural Earth (http://www.naturalearthdata.com/, a free GIS data source) and Africa Open data (https://africaopendata.org/dataset/kenya-counties-shapefile, license Creative Commons) (51).

Of the 1,500 serum samples from cattle, goat, and sheep used for blind virus isolation, one serum sample obtained from a 2- to 3-year-old cow with symptoms of emaciation and lethargy located in Kaptombes village in Kapkuikui, Baringo County (Fig. 1), induced a cytopathic effect (CPE) between 4 and 6 days postinfection (dpi). At the onset, the CPE is characterized by focal degeneration of cells at specific locations within the monolayer. The infected cells become enlarged, rounded, and detach from the surface. The CPE was consistent in four further passages.

### The genome of Kaptombes virus consists of 10 segments of dsRNA.

Infectious cell culture supernatant was applied to high-throughput sequencing (HTS) revealing the genome of an orbivirus with 10 segments of double-stranded (ds) RNA (Table 1). The total genome consisted of 18,731 bp with segment (S) sizes ranging from 3,987 bp (S1) to 781 bp (S10), each predicted to encode a single protein (Table 1). Sequence comparison to sequences available in the NCBI database using BLAST revealed highest similarity of the detected virus to Sathuvachari virus (SVIV), a mosquito-borne orbivirus, which was first isolated from a starling bird (*Brahminy myna*) in India in 1963, and later found in healthy cattle from Japan in 2005 (10, 13) (Table 1). S1, predicted to encode the RNA-dependent RNA polymerase (VP1), showed 77.5% nucleotide identity to the VP1 of SVIV. Similar as observed in mosquito- and tick-borne orbiviruses, S2 is predicted to encode the inner capsid protein VP3 (T2) which showed 80.7% nucleotide identity to the VP3 of SVIV. Segments S3, S4, S5, S6, S7, S8, S9, and S10 of the isolated virus were predicted to encode VP2, VP4, NS1, VP5, NS2, VP7, VP6, and NS3 proteins, respectively, like other mosquito-borne orbiviruses. The VP7 protein also showed highest nucleotide identity of 83.64% to SVIV, which forms the outer core surface and is encoded by S8. The outer capsid protein VP2, encoded by S3, had the least nucleotide identity of 73.3% to that of SVIV.

**TABLE 1:** Comparison of KPTV genome segments and proteins with SVIV (KC432629 to KC432638) and BTV Serotype 1 (FJ969719 to FJ969728)

	Encoded proteins			Segment size (bp)			Protein size (No. of amino acids)			Pairwise identity of KPTV and SVIV	
Segments	KPTV	SVIV	BTV-1	KPTV	SVIV	BTV-1	KPTV	SVIV	BTV-1	nt identity (%)	aa identity (%)
Seg.1	VP1 (Pol)	VP1 (Pol)	VP1 (Pol)	3987	4015	3944	1320	1321	1302	77.46	89.5
Seg.2	VP3 (T2)	VP3 (T2)	VP2 (OC1)	2843	2860	2953	924	919	956	80.71	94.44
Seg.3	VP2 (OC)	VP2 (OC1)	VP3 (T2)	2367	2393	2772	776	777	901	73.73	80.03
Seg.4	VP4 (Cap)	VP4 (Cap)	VP4 (Cap)	2011	1997	1980	641	644	644	77.36	89.43
Seg.5	NS1 (TuP)	NS1 (TuP)	VP5 (OC2)	1788	1789	1769	559	562	552	78.05	87.63
Seg.6	VP5(OC2)	VP5(OC2)	VP6 (Hel)	1622	1644	1638	525	522	526	80.89	93.68
Seg.7	NS2 (ViP)	NS2 (ViP)	VP7 (T13)	1167	1205	1156	371	366	349	81.03	92.08
Seg.8	VP7 (T13)	VP7 (T13)	NS1 (TuP)	1155	1188	1125	351	351	354	83.64	98.29
Seg.9	VP6 (Hel)	VP6 (Hel)	NS2 (ViP)	1010	933	1049	281	287	329	75.60	60.14
Seg.10	NS3	NS3	NS3	781	810	822	213	213	229	82.4	91.04
Total				18,731	18,834	19,208	5961	5962	6142		

**FIG 2:**
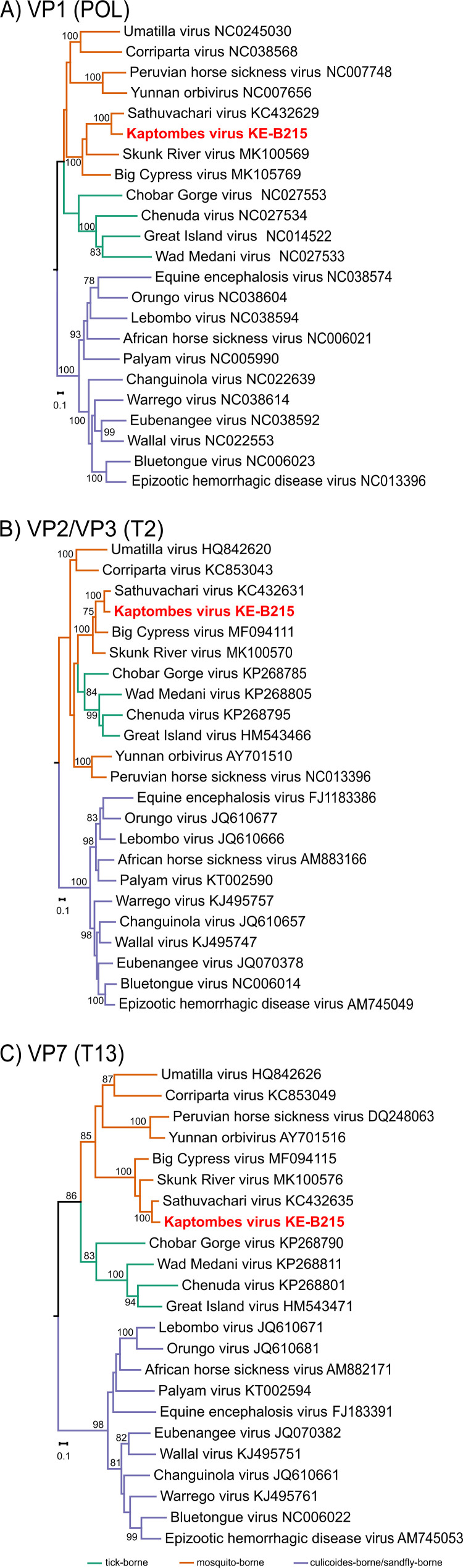
Maximum likelihood phylogenetic analysis of KPTV. Trees were based on the RdRp (Pol 1) (A) the VP3 (T2) (B) and the VP7 (T13) (C) proteins. Sequences were aligned using MAFFT and trees inferred using IQ-Tree 2 and GTR+F+I+G4 (VP1), GTR+F+R5 (T2) and TVM+F+R4 (VP7) substitution models employing 1000 bootstrap replicates.

According to the orbivirus sequence related species demarcation criteria of the ICTV, distinct species share less than 78% amino acid identity in their RdRp proteins and less than 83% amino acid identity (>76% nucleotide identity) in their VP3 protein. The closest genetic relative of KPTV was SVIV with nucleotide (nt) and amino acid (aa) identities of 77.46% and 89.5%, as well as 80.71% and 94.44% in their VP1 (RdRp) and VP3 (T2) proteins, respectively, suggesting that both viruses may belong to the same virus species. The orbivirus outer-core VP7 (T13) is more conserved than the outer-capsid proteins, VP2 (OC1) and VP5 (OC2), and is considered virus-species specific and therefore targeted for serological assays (12, 15, 30). VP7 of KPTV showed nucleotide and amino acid identities of 83.64% and 98.29% to SVIV VP7 (strain IAn-66411). Phylogenetic analyses based on KPTV VP1 (Pol), VP3 (T2), and VP7 (T13) protein sequences showed that KPTV was placed in basal relationship to SVIV sequences and that the genetic distance of KPTV to all SVIV sequences was greater than that within the SVIV clade clearly separating the African KPTV from its Asian relative (Fig. 2).

**FIG 3:**
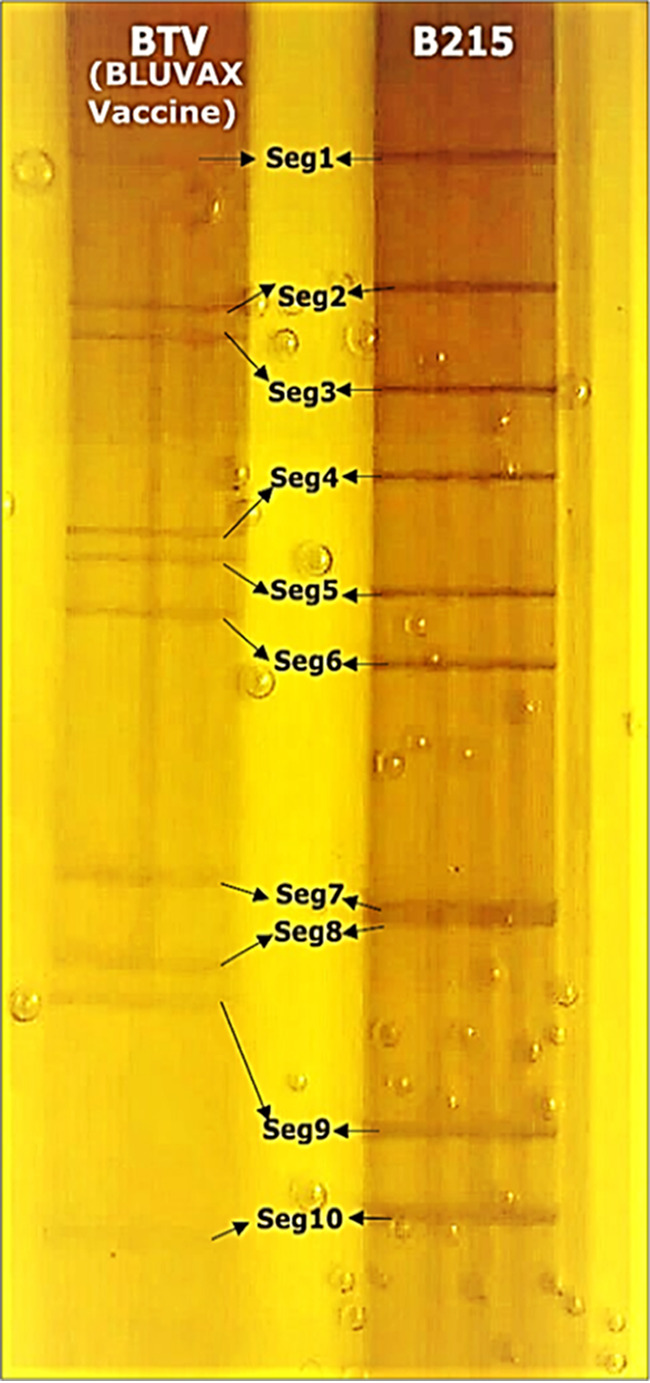
Electrophoretic profile of dsRNA of KPTV and BTV. Viral RNA of KPTV and BTV was separated on a 10% SDS-PAGE gel and stained with silver nitrate.

An SDS-PAGE analysis confirmed the presence of 10 dsRNA segments of different sizes in KPTV and in the BTV BLUVAX vaccine strain, which was used as a control (Fig. 3). Genome segments of KPTV generated a 6-2-2 migration pattern: all genome segments migrated separately, except for S7 and S8, which are almost equal in size and comigrated in the gel system. In contrast, the BTV BLUVAX vaccine strain showed a 3-3-3-1 migration pattern.

### Kaptombes virus infects diverse vertebrate and mosquito cell lines.

**FIG 4:**
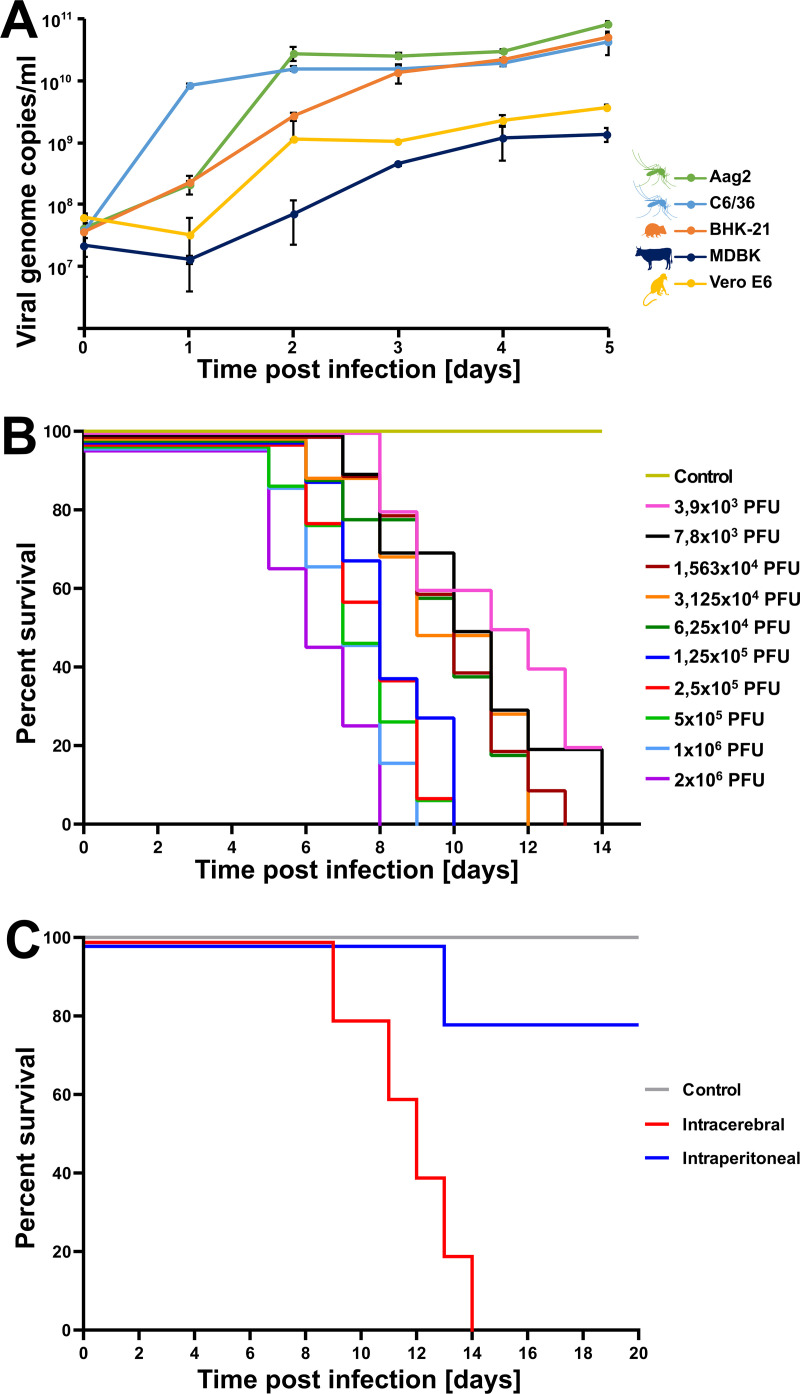
*In vivo* and *in vitro* growth characteristics of KPTV. (A) C6/36, Aag-2, BHK-21, MDBK, and VeroE6 cell lines were infected with KPTV in duplicates at an MOI of 0.1 and supernatant was harvested and quantified by real-time RT-PCR every 24 h for a period of 5 days. (B) Two-fold serial dilutions (1:2 to 1:512) of KPTV were intracerebrally inoculated into 3- to 4-day-old Swiss Albino suckling mice and observed 2 times/day for up to 14 days for signs of disease. (C) Swiss Albino mice (3 to 4 weeks old) were injected intraperitoneally (IP) and intracerebrally (IC) in groups of five with 500 μL of the virus inoculum at a concentration of 1.2 × 10^6^ diluted in PBS. Control mice were injected with equal amount of PBS. All mice were observed daily for 20 days for presentation of symptoms.

KPTV cell tropism was analyzed using bovine (MDBK), primate (Vero E6), and rodent (BHK 21) cells, as well as the mosquito cell lines C6/36 and Aag2. All cell lines were broadly susceptible for KPTV infection with peak genome copy numbers in cells derived from mosquitoes and hamster (Fig. 4A). KPTV reached peak genome copy numbers of ca. 3 × 10^10^ copies/mL at 2- and 3-dpi in mosquito- and rodent-derived cell lines. In contrast, peak genome copies were about 10-fold lower and reached at 4 dpi in cell lines derived from cattle and nonhuman primates. These findings, together with the phylogenetic grouping within the mosquito-borne orbiviruses, suggest a mosquito and vertebrate transmission cycle for KPTV, similar to those of other mosquito-borne orbiviruses like SVIV, Umatilla virus and Skunk River Virus among others (6, 13, 15, 31).

### Kaptombes virus is pathogenic to Swiss Albino mice.

New-born Swiss Albino suckling mice were infected with a range of 10 doses of KPTV (2 × 10^6^, 1 × 10^6^, 5 × 10^5^, 2.5 × 10^5^, 1.25 × 10^5^, 6.25 × 10^4^, 3.125 × 10^4^, 1.563 × 10^4^, 7.8 × 10^3^, and 3.9 × 10^3^ PFU/mL) or mock-infected via intracerebral inoculation in groups of 10. Intracerebral inoculation of KPTV into 3- to 4-day-old Swiss Albino suckling mice resulted in severe clinical disease and induced tremors, hind-limb paralysis, and prostration. None of the clinically diseased mice recovered and lethal outcome was observed 1 to 7 days after onset of symptoms (Fig. 4B; Table S1). Two mice infected with the lowest KPTV dose did not develop symptoms of disease until the end of the experiment. Onset of symptom presentation and time to death was observed 4 to 5 dpi and 1 to 4 days after onset of symptoms in mice infected with highest virus concentrations (2 × 10^6^, 1 × 10^6^ PFU/mL), respectively. All 10 mice of both study groups infected with the highest virus concentrations succumbed to the infection by day 8 and 9. Onset of symptom presentation and lethal outcome after onset of symptoms was observed up to 6 and up to 3 days later, respectively, in mice infected with lower KPTV doses (7.8 × 10^3^, and 3.9 × 10^3^ PFU/mL) compared to mice infected with higher virus doses (2 × 10^6^, 1 × 10^6^ PFU/mL). All infected mice exhibited high infectious virus concentrations in the brain of 2.9 × 10^6^ PFU/mL (mean virus titer).

10.1128/msphere.00488-22.1 TABLE S1 *In vivo* pathogenicity study in Swiss Albino mice. Download Table S1, DOCX file, 0.02 MB. Copyright © 2023 Omoga et al. 2023 Omoga et al. https://creativecommons.org/licenses/by/4.0/ This content is distributed under the terms of the Creative Commons Attribution 4.0 International license.

Furthermore, 3- to 4-week-old Swiss Albino mice in groups of five were either injected intraperitoneally or intracerebrally with 1.2 × 10^6^ PFU/mL of KPTV. All five intracerebrally injected 3- to 4-week-old Swiss mice died within 14 dpi after exhibiting body tremors, hind limb paralysis, general weakness, and lethargy starting from 7 dpi. In contrast, lethal outcome was rare in adult mice infected intraperitoneally. Only one of the intraperitoneally injected mice developed symptoms from day 9 and died 14 dpi (Fig. 4C). The other four intraperitoneally injected mice did not develop any signs of disease. Infectious KPTV particles were recovered from the brain tissues of the deceased animals with a mean titer of 4.2 × 10^4^ PFU/mL. No signs of disease and no mortality were observed in any of the control mice during this study.

### Kaptombes virus infects cattle, goat, and sheep from Baringo county.

**FIG 5:**
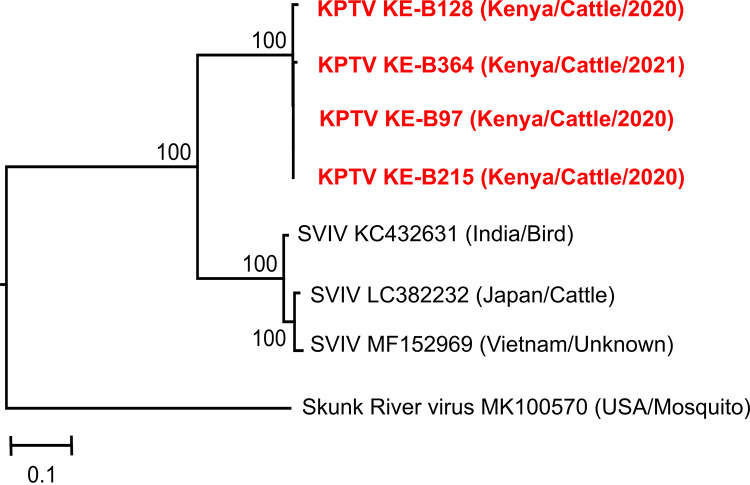
Phylogenetic relationship of KPTV strains. The maximum likelihood tree is based on a 735 nt fragment of the VP3 (T2) gene and was inferred using IQ-TREE 2 and a nonparametric bootstrapping with 1000 replicates. Sequences identified in this study are highlighted in red.

KPTV RNA was detected in three additional samples from asymptomatic cattle (1.2%, 3/248) collected at different locations in 2020 and 2021 (Table 2). Phylogenetic analyses based on obtained KPTV sequence fragments of 735 nucleotide (nt) in size revealed that the Kenyan sequences (KE-B215-2020, KE-B97-2020, KE-B364-2021, and KE-B128-2020) formed a well-supported sister clade to all SVIV sequences (Fig. 5).

**TABLE 2:** KPTV positive livestock samples

Sample ID	Origin	Species	Age years	Sex	Yr of collection
KPTV KE-B97	Loropilli	Cattle	1 to 2	Female	2020
KPTV KE-B128	Keper	Cattle	2 to 3	Male	2020
KPTV KE-B215	Kaptombes	Cattle	2 to 3	Female	2020
KPTV KE-B364	Salabani	Cattle	2 to 3	Male	2021

To further assess the dispersal of KPTV, we selected randomly 200 livestock serum samples (cattle *n* = 100, sheep *n* = 50, and goats *n* = 50) from the same geographical area where KPTV originated and screened them by plaque reduction neutralization test (PRNT) for neutralizing reactivity against KPTV. In total, 12 samples (12/200, 6% 95% CI = 3.14 to 10.25) neutralized the virus with titers ranging from 1:20 to 1:320 (cattle 10% [10/100], goats 2% [1/50], and sheep 2% [1/50]) (Table 3). The presence of neutralizing antibodies was highest in cattle (5%, 10/200) compared to both sheep and goats (1%, 2/200), a significant difference in exposure between cattle and small ruminants (Fisher exact test odds ratio [OR] 5.2, 95% 1.13 to 24.1; *P* = 0.035). All antibody positive samples were negative for KPTV RNA by qPCR and none of the PCR positive samples contained KPTV neutralizing antibodies.

**TABLE 3:** KPTV seropositive samples from Baringo County

S. No	Sample ID	Livestock Species	Origin (Village)	Yr of sample collection	Sex M/F	Age (Yrs)	PRNT results				
							1:20	1:40	1:80	1:160	1:320
1	030/B/09	Cattle	Ilngarua	2020	M	2	+	−	−	−	−
2	030/B/42	Cattle	Perkerra	2020	F	2	+	+	+	−	−
3	030/B/71	Cattle	R14	2020	M	1	+	+	−	−	−
4	030/B/83	Cattle	Loropilli	2020	F	2	+	+	+	−	−
5	030/B/107	Cattle	Loropilli	2020	F	3	+	+	−	−	−
6	030/B/111	Cattle	Keper	2020	F	3	+	+	+	+	+
7	030/B/206	Cattle	Kaptombes	2020	M	3	+	+	+	−	−
8	030/B/250	Cattle	Tuwetie	2020	F	3	+	+	+	+	−
9	030/B/326	Cattle	Sirata	2021	F	2	+	−	−	−	−
10	030/B/359	Cattle	Salabani	2021	M	3	+	+	+	−	−
11	030/O/376	Sheep	Kapkuikui	2021	F	3	+	−	−	−	−
12	030/C/388	Goat	Kapkuikui	2021	F	2	+	+	−	−	−

## DISCUSSION

We report the detection and isolation of a novel mosquito-borne orbivirus designated Kaptombes virus from cattle in Kenya. KPTV was initially isolated from a bovine with clinical symptoms aged 2 to 3 years. The virus was subsequently also detected in two cows sampled in the same year in other animal herds from the nearby villages and in another cow within the same subcounty, roughly 70 km away sampled 1 year later. Neutralizing antibodies against KPTV were detected in cattle (10%) and to a small extend in small ruminants (1%). *In vivo* infection experiments with new-born mice induced body tremors, hind limb paralysis, weakness, lethargy, and mortality as from 4 to 5 dpi. Taken together, these findings provide evidence of a novel virus infecting ruminants in Baringo county, Kenya.

An aliquot of the serum sample was sent to the Central Veterinary Laboratory (CVL) Kabete, Kenya for routine diagnostics where no positive results were obtained for tested diseases, including Rift Valley fever (CVL, Lab report). At this time, we cannot confirm the orbivirus infection to be the cause nor dismiss its contribution to the clinical state and symptoms of the animal. The other animals from which the virus was later detected in serum samples showed no symptoms of disease.

In the present study, KPTV RNA was only detected in cattle and not in other livestock species (sheep and goat) screened. However, neutralizing antibodies were also detected in sheep and goats, although with a significant lower prevalence than in cattle. Our data support the previous detection of its closest relative, SVIV in cattle, signifying a probable higher susceptibility of cattle to the virus or a preferred host range to large ruminants (10). An overall seroprevalence of 6% (12/200, 95% CI = 3.14 to 10.25) was found with a significantly higher exposure of cattle to KPTV at 10% compared to 1% in small ruminants (goats and sheep), respectively (*P* = 0.035). Orbivirus specificity to a certain vertebrate host is common and has been observed among some host species, notably, horses which are known to be more susceptible to AHSV or sheep that are more susceptible to BTV. However, these AHSV and BTV can also infect many other host species (20–22). This could also be the case for KPTV implying that cattle may be more susceptible to the virus than sheep and goats. Nonetheless, we recommend further studies to understand its pathogenicity and susceptibility of various livestock species to KPTV. In addition, a larger sample size from different parts of the country will be required to assess distribution and prevalence rates of KPTV.

KPTV’s closest relative is the mosquito-borne SVIV orbivirus, which was first isolated in 1963 from a starling bird (*Brahminy myna*) in India, and later isolated from sera of healthy cattle from Japan in 2005 (10, 13). SVIV has also been isolated from a pool of mosquitoes collected in 1966 in Vietnam (32). The KPTV 6-2-2 dsRNA segment migration pattern in electrophoretic analysis (Fig. 3), is consistent to that of SVIV as presented by Kato et al. (10) indicating genetic relationship of the two viruses. According to the 10th report of the ICTV for orbivirus classification, viruses of the same species have more than 76% nt identity and 83% amino acid (aa) identity in their major sub core structural protein VP3 (T2) and more than 78% aa identity in their VP1 (Pol) proteins. Amino acid sequence identities of 94% and 89,5% between KPTV and SVIV in the T2 and polymerase proteins were found, respectively, suggesting that both viruses may belong to the same species (1, 31, 33). However, the nt and aa identities of the VP2 (OC1) protein to that of SVIV revealed a high genetic variability of 73.73% and 80.3%, respectively (34). Other species demarcation criteria, such as serological cross-reactivity in conserved antigens and cross-hybridization of conserved genome segments, could not be investigated as no SVIV isolate was available. Currently, all SVIV strains are considered to belong to the same serotype due to high sequence identities (97.49%nt/98.58%aa) in the VP2 (OC1) protein (10). Of note, the KPTV VP2 shows a much lower sequence identity of 73.73%nt/80.03% aa, respectively, to that of SVIV and thus may be serologically distinct. Further, KPTV is well separated from all currently known SVIV sequences in phylogenetic analyses supporting the existence of two different genetic lineages or may even indicate the presence of two different species. If KPTV and SVIV share common vector and host species needs further studies. Also, the pathogenicity of the two viruses is currently not well understood. KPTV was found to cause signs of disease and death in new-born and adult mice. Similarly, SVIV was also shown to be pathogenic for new-borne mice (32), though the cattle from which it was isolated in Japan was apparently healthy. Together with the finding that KPTV was isolated from an emaciated and lethargic animal, its pathogenetic potential deserves further studies.

Orbiviruses are known to have noncoding regions (NCRs) at the termini of all segments which vary depending on the virus. Mosquito-borne orbiviruses have an overall NCRs of 5.03% to 5.695% (13, 15). The overall NCRs in the entire KPTV genome was with 4.675%, similar to that of SVIV IAn-66411 with 4.874%. Although for both viruses transmission by mosquitoes is hypothesized, the range of NCRs is less than the reported range for other mosquito-borne orbiviruses. However, the NCRs percentages are within the range for tick-borne orbiviruses of 4.47% to 4.9%, which could be attributed to a common ancestry or other unknown factors (6, 15, 16). Orbiviruses exhibit varied G+C contents based on the transmitting arthropod vector. The documented range for mosquito-borne orbiviruses is between 36.7% in PHSV to 45.55% in YUOV (15). The G+C content of SVIV is 42.3% (13). The tick-borne orbiviruses generally exhibit a higher G+C content of over 50% up to 58.1% in Great island virus (16), while the *Culicoides*/sandfly-borne orbiviruses have an intermediate G+C content of 39.89% to 45.89% (15, 16, 35). KPTV has a G+C content of 43.14%, thus falling within the range of mosquito-borne orbiviruses. Phylogenetic analyses based on VP1 (Pol), VP3 (T2), and VP7 (T13) proteins placed KPTV in the same cluster with mosquito-borne orbiviruses. Mosquito cell lines were found to be susceptible for infection with KPTV further supporting the idea of a mosquito transmission cycle.

SVIV has so far only been reported in three Asian countries (India, Japan, and Vietnam) (10, 13, 32). The occurrence of a related virus, KPTV in Kenya raises questions about the evolutionary origin of the two viruses and their spatial and temporal spread, possibly through movement of host species like migratory birds. SVIV was first detected in birds (13), and annual north-south bird migration/movement trends provide an opportunity for a possible spread of the virus to other geographical regions (36–38). KPTV was detected in Baringo county, which is home to two freshwater lakes (Lake Bogoria and Lake Baringo) that host large populations of diverse migratory bird species. Additional genome sequences are needed to understand the spatial and temporal spread of KPTV and its relatives in Africa and Asia.

### Conclusion.

We report the identification of a novel presumably disease-causing orbivirus in cattle. These findings expand our knowledge of circulating orbiviruses in livestock. Cattle represents one of the most important livestock species in farming industry and often is the main source of livelihoods in rural areas of Africa (39). Thus, infectious diseases of cattle may have major economic consequences and are of high relevance. The data presented here form a baseline for further research, including active surveillance of KPTV to understand its geographic distribution, susceptible vertebrate hosts, pathology, mode of transmission, its potential to cause outbreaks, and its implication for animal or human health.

## MATERIALS AND METHODS

### Livestock sample collection.

Serum samples were collected from randomly selected cattle, sheep, and goats between 2020 and 2022, twice a year in May/June and September/October after the short and long rains in the semiarid areas of Marigat, Baringo County (0.4695° N, 35.9833° E) and Nguruman, Kajiado County (1.7617° S, 36.0255° E) in Kenya. Serum was collected and transported in cold chain (dry ice) to the Martin Lüscher Emerging Infectious Diseases Laboratory at *icipe*.

### Virus isolation in cell culture.

Vero CCL-81 (ATCC CCL-81) cells were seeded in 24-well tissue culture plates (Nunc, Roskilde, Denmark) to 80% confluence in Gibco Dulbecco’s modified Eagle’s medium (DMEM) containing 10% Gibco fetal bovine serum (FBS), 2% Gibco l-glutamine (200 mM), 2% Gibco antibiotic-antimycotic (100×). The cells were rinsed with Gibco PBS, pH 7.4, and 50 μL serum was added followed by incubation at 37°C, 5% CO_2_ (New Brunswick Galaxy 170 R CO_2_ Incubator Series, Eppendorf, USA) for 1 h, rocking after every 15 min to allow virus adsorption. After incubation, Gibco DMEM maintenance medium (MM) with 5% Gibco FBS, 2% Gibco l-glutamine (200 mM), and 2% Gibco Antibiotic-Antimycotic (100×) was added. Cells were incubated at 37°C, 5% CO_2_ (New Brunswick Galaxy 170 R CO_2_ Incubator Series, Eppendorf, USA) and observed daily for signs of cytopathic effects (CPE). The CPE positive sample was passaged in 25-cm^2^ cell culture flasks (Nunc, Roskilde, Denmark) and frozen at –80°C before harvesting by thawing and centrifuging at 3,000 rpm for 10 min. The infectious supernatant was stored at –80°C until further use.

### Next-generation sequencing.

Clarified infectious cell culture supernatant was filtered using 0.22-μm filters (Merck Millipore Co., MA, USA) to remove possible cellular residues. RNA was extracted using QIAamp viral RNA minikit (Qiagen, Hilden Germany) following the manufacturer’s protocol. RNA was quantified using a Nanodrop 2000 spectrophotometer (Thermo Fisher Scientific, USA) and Qubit RNA 2.0 fluorometer using the Qubit RNA HS assay kit (Invitrogen, USA). Libraries for sequencing were prepared using TruSeq stranded mRNA kit (Illumina, USA), following the manufacturer’s protocol with the modification to exclude the poly(A)-containing mRNA purification steps. Reverse transcription was done using Superscript III reverse transcriptase (Invitrogen, USA) and random hexanucleotide primers (Invitrogen, USA). This was followed by second-strand synthesis using DNA polymerase I and RNase H, provided with the library preparation kit. Purification was performed using AMPure XP beads (Beckman Coulter, USA) after which the purified double-strand cDNA fragments were end repaired by adding a single A’ nucleotides to the 3′ end of the blunt fragments. Ligation of the adapters was performed, and the products purified and enriched by PCR to create the final library. Libraries were normalized, pooled, and sequenced using the Illumina platform.

### *In vitro* viral growth kinetics.

Cell lines derived from insects (Aedes albopictus C6/36, *Aedes aegytpi* Aag-2) and vertebrates (hamster, BHK-21; bovine, MDBK; primate, VeroE6) were infected with KPTV in duplicates at a multiplicity of infection (MOI) of 0.1. Aliquots of infectious cell culture supernatants from vertebrate and insect cells were harvested every 24 h for a period of 5 days. Amount of viral genome copy numbers were quantified by real-time RT-PCR using plasmid-based quantification standards (40–42).

### *In vivo* pathogenicity studies in mice.

Swiss Albino mice obtained from the KEMRI animal unit were used. Eleven female lactating mice with a litter size of 10 were selected for the study and the neonates observed for 2 to 3 days before the experiment in a Biosafety Level 2 (BSL 2) laboratory. Mice were fed *ad libitum*. One-hundred microliters of the third passage of KPTV, as well as nine consecutive 2-fold serial dilutions (1:2 to 1:512) of it were intracerebrally inoculated through the cranial wall of the skull into 3- to 4-day-old Swiss Albino suckling mice. All 10 pups of one litter in a cage were infected with the same virus dose. The doses used in the experimental infection were quantified by plaque assays in Vero CCL-81 cells and the corresponding viral titers were as follows: 2 × 10^6^, 1 × 10^6^, 5 × 10^5^, 2.5 × 10^5^, 1.25 × 10^5^, 6.25 × 10^4^, 3.125 × 10^4^, 1.563 × 10^4^, 7.8 × 10^3^, 3.9 × 10^3^ PFU/mL. Noninfectious MEM was included as negative control. All mice were observed 2 times/day for up to 14 days for signs of infection and any severely moribund mice separated and preserved at –80°C immediately after death. Brain samples were homogenized in 1 mL of cell culture media and inoculated in Vero CCL-81 cells. Infectious viral particles were quantified via plague assay.

Adult 3- to 4-week-old Swiss Albino mice in groups of five were injected intraperitoneally (IP) on the lower abdomen through the abdominal wall and intracerebrally (IC) with 500 μL of the virus inoculum at a concentration of 1.2 × 10^6^ PFU/mL diluted in phosphate-buffered saline (PBS, pH 7.5). Control mice were injected with equal amount of PBS. Infected mice were observed daily for 20 days for specific symptoms presentation (SSP) (43, 44).

### Sodium dodecyl sulphate–polyacrylamide gel electrophoresis (SDS-PAGE).

**(i) RNA extraction.** For dsRNA segment analyses, 50 μL of prewarmed 1M sodium acetate (NaAc) containing 1% sodium dodecyl sulfate (SDS), pH 5.0 was added to 450 μL of infectious cell culture supernatant from either KPTV or the BTV BLUVAX vaccine from KEVEVAPI (Kenya Veterinary Vaccine Production Institute) isolate, vortexed for 10 s and incubated for 15 min at 37°C. Next, 500 microliters phenol-chloroform (1:1) was added to the tube, vortexed for 1 min then incubated for 15 min at 56°C in a water bath. After incubation, the sample was vortexed for 1 min then centrifuged for 5 min at 12,000 rpm. The upper aqueous phase containing the dsRNA was pipetted into a clean Eppendorf tube and 40 μL (~1/10 volume) 3M NaAc added and the tube filled with ice-cold (–20°C) absolute ethanol, mixed gently by turning the tube over and over 4 to 6 times then incubated at –20°C overnight. The tubes were centrifuged at 4°C for 20 min at 12,000 rpm to pellet the dsRNA, the supernatant was poured off and tubes were air dried. The pellet was resuspended in 30 μL PAGE sample dye containing 10 mg bromophenol blue, 5 mL of Spacer gel buffer, and 1 mL of glycerol before loading on a PAGE gel.

**(ii) PAGE analysis.** A total of 10%1.5 mm Resolving Gel was prepared using 15.8 mL distilled water, 10 mL of 30% acrylamide stock, 3.75 mL resolving buffer (pH 8.9) containing 36.3 g Tris Base and 48 mL 1N HCL, 15 μL Tetramethylethylenediamine (TEMED), and 450 μL of 10% ammonium persulphate. The gel was immediately poured between the thick gel spacers up to the gel mark after cleaning with 96% ethanol then overlaid with 1 mL layer of water before polymerization. The resolving gel was allowed to polymerise for 2 h, after which the water was poured off and 1.5 mm 3% spacer gel prepared by 6.8 mL distilled water, 1.6 mL of 30% acrylamide stock, 1.25 mL spacer buffer (pH 6.7) containing 5.98 g Tris Base and 50 mL distilled water, 5 μL TEMED, and 150 μL of 10% ammonium persulphate was poured on top after positioning the combs and allowed to polymerize for 45 min. After polymerization, the combs were removed, and the glass plate assembled onto the electrophoresis apparatus with electrophoresis buffer prepared by 200 mL 5% Tris-glycerine buffer with 800 mL of distilled water. The dsRNA of KPTV and BTV BLUVAX vaccine extracted as earlier described and diluted in 30 μL PAGE-dye was loaded to the wells and run for 18 h at 100V.

After electrophoresis, the gel was washed and fixed with 200 mL fixing solution 1, prepared with 80 mL ethanol, 110 mL distilled water, and 10 mL acetic acid then incubated for 30 min on an orbital shaker. The fixing solution 1 was drained off and replaced with 200 mL of fixing solution 2 containing 20 mL ethanol, 180 mL distilled water, and 1 mL acetic acid and incubated for 30 min on the orbital shaker. The fixing solution 2 was drained off and the gel stained for 30 min using silver nitrate (AgNO_3_) staining solution then washed twice in distilled water for 2 min each wash. To remove the black precipitate, approximately 50 mL of developing solution (7.5g NaOH, 250 mL dH_2_O, 2 mL 36% Formaldehyde) was used, agitated for 30 s, poured off then 200 mL added, and incubated for 5 min, drained off before adding stopping solution (10 mL acetic acid and 200 mL dH_2_O) to prevent further color development for 10 min and rinsing the gel in distilled water for 10 min. Distilled water was added to the gels and left until ready to dry.

### Primer design, PCR screening, and Sanger sequencing.

KPTV specific primers (F 5′-AGCGAGGTGGATAGTGAAGA-3′ and R 5′-CTCCGCCCTAACATCCAATAAA-3′) and real-time RT-PCR primers (F 5′-TTGGGACGGAAGCGACTTAG-3′, R 5′-ATCTCCTCCTGCATGACACG-3′ and probe FAM/AAACTCTAC/ZEN/TCTGAT CGCAAATTCG/3IABkFQ) were designed based on Segment 2 using the IDT PrimerQuest Tool.

A total of 248 livestock serum pools made up of 83/248 (33.5%) cattle, 85/248 (34.3%) goats, and 80/248 (32.2%) sheep from Baringo county were screened for presence of KPTV RNA. Each pool consisted of five to seven individual samples based on species and location. Viral RNA was extracted from 140 μL pooled serum and the CPE positive sample (KPTV) using the QIAamp Viral RNA minikit (Qiagen, Hilden Germany) according to the manufacturer’s protocol. cDNA was synthesized using SuperScript III Reverse Transcriptase according to the manufacturer’s instructions. The samples were tested for presence of KPTV genome copies using a 25 μL PCR containing 15.65 μL PCR water, 2.50 μL 10 × PCR Buffer, 1.25 μL Mg (50 mM), 0.50 μL 10 mM dNTPs, 1.5 μL of 10 μM forward and reverse primers, 0.10 μL Platinum-*Taq* polymerase, and 2.0 μL cDNA template. Cycling conditions were 95°C for 3 min, followed by 40 cycles of 95°C for 15 s, 55°C for 40 s, and 72°C for 1 min and a further extension of 72°C for 10 min then 4°C for infinity. The PCR products were electrophoresed in 2% agarose gel stained with ethidium bromide (Sigma-Aldrich Chemie GmbH) and positive samples purified using ExoSAP-IT PCR Product Clean-up Reagent (Applied Biosystems) according to the manufacturer’s instructions, then sequenced in both directions. The sequencing services were outsourced to Macrogen, Europe B.V.

The 25 μL qRT-PCR total reaction volume included 16.05 μL of water, 0.75 μL forward and reverse primer each, 0.25 μL FAM^T^ target probe, 2 μL Mg (50 mM), 2.5 μL 10 × Buffer, 0.5 μL dNTPS (10 mM), 0.2 μL Invitrogen Platinum *Taq*, and 2 μL cDNA template.

### Screening for KPTV neutralizing antibodies using PRNT.

A total of 200 livestock samples from Baringo county (cattle *n* = 100, sheep *n* = 50, and goats *n* = 50) were aliquoted in volumes of 30 μL, heat inactivated at 56°C for 30 min and then tested for neutralizing antibodies to Kaptombes virus in 2-fold serum dilutions from 1:20 to 1:320 using PRNT_90_ as previously described (41, 45, 46).

### Data management and analysis.

**(i) Seroprevalence, *in vivo,* and *in vitro* data.** The seroprevalence, *in vivo*, and *in vitro* studies data were entered into Microsoft Excel v. 2016, cleaned then imported to R version 4.2.0 for analysis. Comparison of KPTV seroprevalence between the different livestock species was done using Fisher exact test. The 95% confidence intervals (CIs) were estimated using the Agresti-Coull method. All tests were performed at a 5% significance level.

**(ii) Sanger sequence and phylogenetic analysis.** Sequences were analyzed using Geneious Prime and queried against the NCBI GenBank database using the Basic Local Alignment Search Tool (BLAST) (47). Multiple sequence alignments were performed using MAFFT (48) and maximum likelihood phylogenetic analyses were performed in IQ-TREE 2 (49), using the GTR+F+I+G4, the GTR+F+R5, and the TVM+F+R4 substitution model for the VP1, T2, and VP7 proteins, respectively, selected by ModelFinder (50). In total, 1,000 bootstrap replicates were applied.

**(iii) NGS data analysis.** Raw sequence reads were initially subjected to cleaning using Trim Galore v0.6.5 to remove adapters and Prinseq Lite v0.20.4 to remove low-quality reads using the following parameters: minimum length, 50 bp; maximum length, 301 bp; and minimum mean Q score, 30. Further, filtering of the reads was performed by using ribo Picker v0.4.3, to remove rRNA sequences by comparing them to the SILVA rRNA database, release 138.1. Paired-end reads were merged using PEAR 0.9.8, and preliminary analysis was performed using the MG-RAST server to identify reads taxonomically. De novo assembly of cleaned reads was done using trinity program with default parameters. The cleaned reads were mapped back to the assembled contigs and filtered to retain only contigs in which at least 90% of nucleotides had a 5-time coverage. Contigs were compared to the NCBI database. Sequences were further analyzed in Geneious Prime software (https://www.geneious.com).

### Ethical consideration.

The study was approved by the Kenya Medical Research Institute’s Scientific and Ethics Review Unit (under SERU No.3312) after gaining approval by the animal care and use committee. Additional approval for the study was accorded by the University of Pretoria, Faculty of Health Science’s Research Ethics Committee (Ethics Reference No. 568/2020).

### Data availability.

The sequences of this study can be found under the GenBank accession numbers KPTV B215 genome OQ122118 to OQ122127, as well as fragments of S2 of KPTV B97, KPTV B215, KPTV B128, and KPTV B364 under the accession numbers OQ122128 to OQ122131, respectively.
