# Supplementary material for: Characterization of a Novel Orbivirus from Cattle Reveals Active Circulation of a Previously Unknown and Pathogenic Orbivirus in Ruminants in Kenya
Source: mSphere. 2023 Feb 16;8(2):e00488-22. doi: 10.1128/msphere.00488-22 (PMC10117150; doi:10.1128/msphere.00488-22)
Supplement: TABLE S1 [file msphere.00488-22-s0001.docx]

**Supplementary Information Table 1:** In vivo pathogenicity study in Swiss Albino mice.

| **Virus Conc**. | **Days post infection (dpi)** | | | | | | | | | | | | | | | | | | | | | | | | | | | | | | | | | | | | | | | | | |
| --- | --- | --- | --- | --- | --- | --- | --- | --- | --- | --- | --- | --- | --- | --- | --- | --- | --- | --- | --- | --- | --- | --- | --- | --- | --- | --- | --- | --- | --- | --- | --- | --- | --- | --- | --- | --- | --- | --- | --- | --- | --- | --- |
|  | **Day 1** | | | **Day 2** | | | **Day 3** | | | **Day 4** | | | **Day 5** | | | **Day 6** | | | **Day 7** | | | **Day 8** | | | **Day 9** | | | **Day 10** | | | **Day 11** | | | **Day 12** | | | **Day 13** | | | **Day 14** | | |
|  | **N** | **S** | **D** | **N** | **S** | **D** | **N** | **S** | **D** | **N** | **S** | **D** | **N** | **S** | **D** | **N** | **S** | **D** | **N** | **S** | **D** | **N** | **S** | **D** | **N** | **S** | **D** | **N** | **S** | **D** | **N** | **S** | **D** | **N** | **S** | **D** | **N** | **S** | **D** | **N** | **S** | **D** |
| **2x10⁶** | 10 | 0 | 0 | 10 | 0 | 0 | 10 | 0 | 0 | 6 | 4 | 0 | 2 | 5 | 3 | 0 | 5 | 5 | 0 | 3 | 7 | 0 | 0 | 10 | - | - | - | - | - | - | - | - | - | - | - | - | - | - | - | - | - | - |
| **1x10⁶** | 10 | 0 | 0 | 10 | 0 | 0 | 10 | 0 | 0 | 8 | 2 | 0 | 5 | 4 | 1 | 0 | 7 | 3 | 0 | 5 | 5 | 0 | 2 | 8 | 0 | 0 | 10 | - | - | - | - | - | - | - | - | - | - | - | - | - | - | - |
| **5x10⁵** | 10 | 0 | 0 | 10 | 0 | 0 | 10 | 0 | 0 | 10 | 0 | 0 | 4 | 5 | 1 | 1 | 7 | 2 | 0 | 5 | 5 | 0 | 3 | 7 | 0 | 1 | 9 | 0 | 0 | 10 | - | - | - | - | - | - | - | - | - | - | - | - |
| **2.5x10⁵** | 10 | 0 | 0 | 10 | 0 | 0 | 10 | 0 | 0 | 10 | 0 | 0 | 6 | 4 | 0 | 3 | 5 | 2 | 1 | 5 | 4 | 0 | 4 | 6 | 0 | 1 | 9 | 0 | 0 | 10 | - | - | - | - | - | - | - | - | - | - | - | - |
| **1.25x10⁵** | 10 | 0 | 0 | 10 | 0 | 0 | 10 | 0 | 0 | 9 | 1 | 0 | 5 | 5 | 0 | 4 | 5 | 1 | 3 | 4 | 3 | 3 | 1 | 6 | 1 | 2 | 7 | 0 | 0 | 10 | - | - | - | - | - | - | - | - | - | - | - | - |
| **6.25x10⁴** | 10 | 0 | 0 | 10 | 0 | 0 | 10 | 0 | 0 | 10 | 0 | 0 | 7 | 3 | 0 | 7 | 2 | 1 | 7 | 1 | 2 | 5 | 3 | 2 | 2 | 4 | 4 | 0 | 4 | 6 | 0 | 2 | 8 | 0 | 0 | 10 | - | - | - | - | - | - |
| **3.125x10⁴** | 10 | 0 | 0 | 10 | 0 | 0 | 10 | 0 | 0 | 10 | 0 | 0 | 8 | 2 | 0 | 8 | 1 | 1 | 8 | 1 | 1 | 7 | 0 | 3 | 3 | 2 | 5 | 0 | 5 | 5 | 0 | 3 | 7 | 0 | 0 | 10 | - | - | - | - | - | - |
| **1.563x10⁴** | 10 | 0 | 0 | 10 | 0 | 0 | 10 | 0 | 0 | 10 | 0 | 0 | 9 | 1 | 0 | 7 | 3 | 0 | 7 | 2 | 1 | 4 | 4 | 2 | 1 | 5 | 4 | 0 | 4 | 6 | 0 | 2 | 8 | 0 | 1 | 9 | 0 | 0 | 10 | - | - | - |
| **7.8x10³** | 10 | 0 | 0 | 10 | 0 | 0 | 10 | 0 | 0 | 10 | 0 | 0 | 10 | 0 | 0 | 9 | 1 | 0 | 9 | 0 | 1 | 4 | 3 | 3 | 2 | 5 | 3 | 1 | 4 | 5 | 0 | 3 | 7 | 0 | 2 | 8 | 0 | 2 | 8 | 0 | 0 | 10 |
| **3.9x10³** | 10 | 0 | 0 | 10 | 0 | 0 | 10 | 0 | 0 | 10 | 0 | 0 | 10 | 0 | 0 | 10 | 0 | 0 | 8 | 2 | 0 | 4 | 4 | 2 | 2 | 4 | 4 | 2 | 4 | 4 | 2 | 3 | 5 | 2 | 2 | 6 | 2 | 0 | 8 | 2 | 0 | 8 |
| **Control** | 10 | 0 | 0 | 10 | 0 | 0 | 10 | 0 | 0 | 10 | 0 | 0 | 10 | 0 | 0 | 10 | 0 | 0 | 10 | 0 | 0 | 10 | 0 | 0 | 10 | 0 | 0 | 10 | 0 | 0 | 10 | 0 | 0 | 10 | 0 | 0 | 10 | 0 | 0 | 10 | 0 | 0 |

Total number of mice **N**: Normal, **S**: Sick, **D**: Dead
